# Technical pitfalls when collecting, cryopreserving, thawing, and stimulating human T-cells

**DOI:** 10.3389/fimmu.2024.1382192

**Published:** 2024-05-15

**Authors:** Daniel J. Browne, Catherine M. Miller, Denise L. Doolan

**Affiliations:** ^1^ Centre for Molecular Therapeutics, Australian Institute of Tropical Health and Medicine, James Cook University, Cairns, QLD, Australia; ^2^ College of Medicine and Dentistry, James Cook University, Cairns, QLD, Australia; ^3^ Institute for Molecular Bioscience, The University of Queensland, St Lucia, QLD, Australia

**Keywords:** PBMC, epitopes, stimulation, human, T cell

## Abstract

The collection, cryopreservation, thawing, and culture of peripheral blood mononuclear cells (PBMCs) can profoundly influence T cell viability and immunogenicity. Gold-standard PBMC processing protocols have been developed by the *Office of HIV/AIDS Network Coordination* (HANC); however, these protocols are not universally observed. Herein, we have explored the current literature assessing how technical variation during PBMC processing can influence cellular viability and T cell immunogenicity, noting inconsistent findings between many of these studies. Amid the mounting concerns over scientific replicability, there is growing acknowledgement that improved methodological rigour and transparent reporting is required to facilitate independent reproducibility. This review highlights that in human T cell studies, this entails adopting stringent standardised operating procedures (SOPs) for PBMC processing. We specifically propose the use of HANC’s *Cross-Network PBMC Processing SOP*, when collecting and cryopreserving PBMCs, and the HANC member network *International Maternal Pediatric Adolescent AIDS Clinical Trials* (IMPAACT) *PBMC Thawing SOP* when thawing PBMCs. These stringent and detailed protocols include comprehensive reporting procedures to document unavoidable technical variations, such as delayed processing times. Additionally, we make further standardisation and reporting recommendations to minimise and document variability during this critical experimental period. This review provides a detailed overview of the challenges inherent to a procedure often considered routine, highlighting the importance of carefully considering each aspect of SOPs for PBMC collection, cryopreservation, thawing, and culture to ensure accurate interpretation and comparison between studies.

## Introduction

T-lymphocytes (T cells) are integral components of adaptive immunity (1), and are essential for clearing infections (2), responding to vaccinations (3) or emergent tumorigenesis (4), and maintaining immune system homeostasis (5). Given these broad and critical effector functions, T cells have been the focus of intensive research which predominantly aims to: i) characterise and compartmentalise T cell phenotypes (6, 7), and ii) understand and modulate T cell immunogenicity (8, 9). T cells can be broadly characterised as either CD4^+^ Helper T cells, or CD8^+^ cytotoxic T cells (1). CD4^+^ T cells are crucial for regulating the immune response by releasing signalling molecules that activate, modulate, or direct other immune cells against a particular pathogen (3, 5). Cytotoxic CD8^+^ T Cells are primarily responsible for killing dangerous self-cells, such as those infected with intracellular pathogens or cells undergoing tumorigenesis (3, 4). Both CD4^+^ and CD8^+^ T cell immunogenicity is commonly studied through functional immunoassays that investigate the activation and behaviour of T cells in response to specific stimuli (10). These experiments generally involve the *in-vitro* stimulation of peripheral blood mononuclear cells (PBMCs) with antigenic peptide epitopes or non-specific mitogens. This mimics the *in-vivo* activation of T cells, which initiates various responses including cytokine production (11), proliferation (12), or apoptosis (13). Optimal assessment of T cell phenotypes and immunogenicity requires PBMCs that are viable, and which retain their natural *in-vivo* immunogenic capabilities.

The *in-vitro* loss of PBMC viability or *in-vivo* T cell immunogenicity can critically impact immunological research and clinical trials (14, 15). A notable example is the differential response of T cells when stimulated either *in-vitro* or *in-vivo* with the CD28 agonist antibody TGN1412 (16). In 2006, a phase I clinical study triggered life threatening cytokine release syndrome in patients infused with TGN1412 (17). Further studies revealed that T-cell activation by TGN1412 was dependent upon co-stimulatory signals (18), which were restored to *in-vivo* conditions *in-vitro* when PBMCs were pre-cultured (rested) in high densities (14, 19). More broadly, persistent controversies in T cell research, such as inconsistencies between vaccine antigen testing and vaccine clinical trial immunogenicity (20–22), disagreements on immunodominant antigen or epitopes (23, 24), or other debated aspects of immunological responses (25), may be partly explainable by technical variation during PBMC processing.

**Figure 1:**
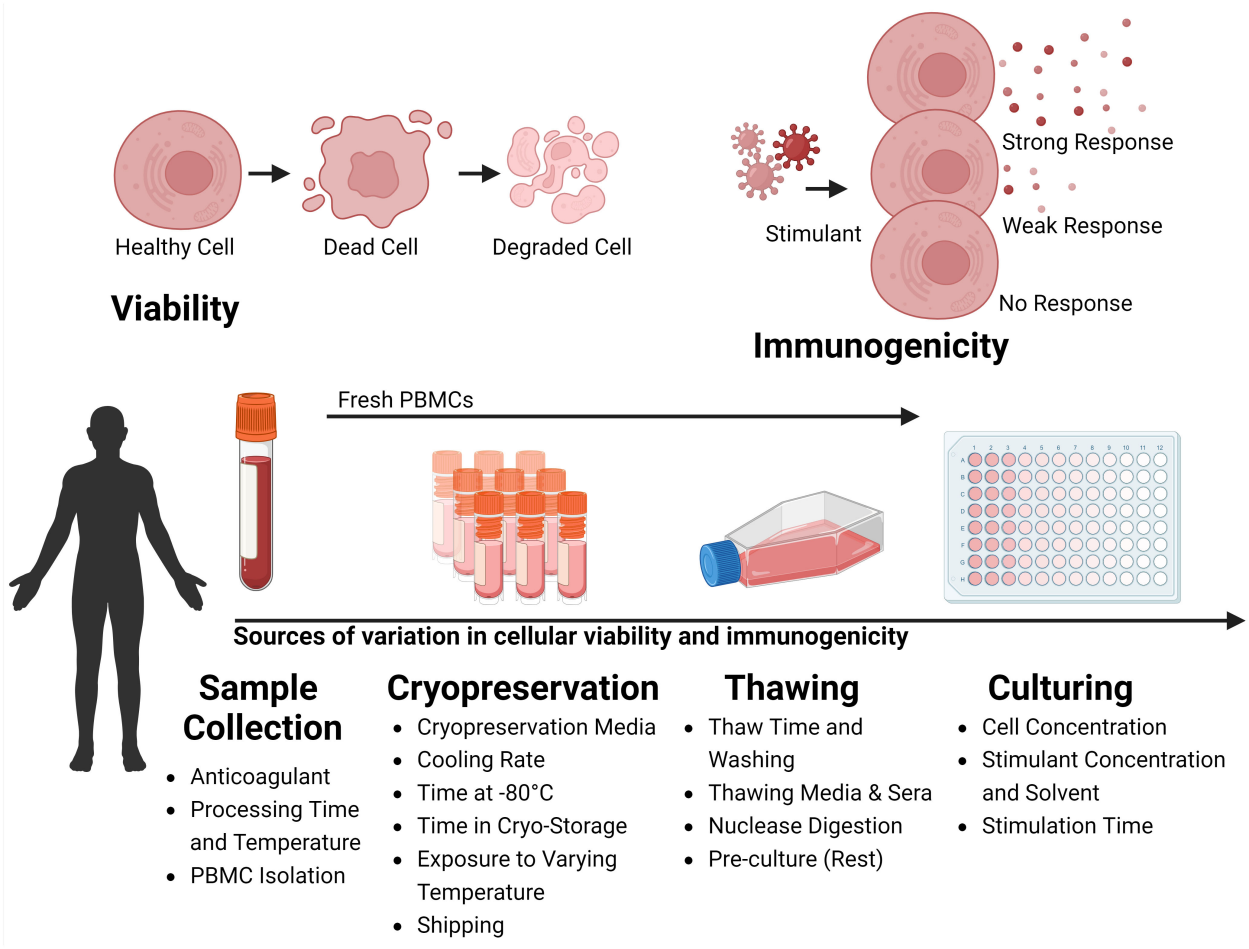
Variability Factors in Cellular Viability and Immunogenicity in Sample Processing. The process of collection, storage, thawing, and culturing cells collected from the peripheral blood of human donors can influence the viability and the immunogenicity of the sample. During Sample Collection, variability arises from the type of anticoagulant used, the time and temperature of processing, and the strategy for isolating Peripheral Blood Mononuclear Cells (PBMCs). During Cryopreservation variability is influenced by the choice of cryopreservation media, cooling rate, duration at -80°C before long-term storage, storage duration, temperature fluctuations during storage, and conditions during shipping. During Thawing, factors include thawing time, wash strategies, thawing media and sera, nuclease digestion, and whether cells are rested before experiments. During Culturing, the cell concentration, stimulant dose, and duration of stimulation affect outcomes. Viability is marked by a transition from healthy to dead cells, increasing cellular debris. Immunogenicity, is the ability of cells to react to stimulants like antigenic peptides or mitogens, is typically assessed through functional immunoassays measuring the immune response via the production of surrogate markers of immunity. Created with BioRender.com.

PBMC processing involves collecting, storing, thawing, and culturing PBMCs from human donors (26). This protocol typically involves: i) collecting peripheral blood using venepuncture, ii) separating PBMCs from other blood components, iii) immediately experimenting on, or cryopreserving the cells, iv) thawing cryopreserved cells, and v) *in-vitro* cell culture. Following the outbreak of the HIV/AIDS epidemic there was a heightened need for a globally coordinated T cell clinical trial network with a standardised PBMC processing protocol (27, 28). In response, the *Office of HIV/AIDS Network Coordination* (HANC) established gold-standard PBMC processing standard operating procedures (SOPs), which were widely, but not universally adopted (29, 30). The current SOPs include the HANC’s *Cross-Network PBMC Processing SOP*, for collecting and cryopreserving PBMCs (31), and the HANC member network *International Maternal Pediatric Adolescent AIDS Clinical Trials* (IMPAACT) *PBMC Thawing SOP* when thawing PBMCs (32). This review will demonstrate even relatively minor deviations from these SOPs can have profound consequences for PBMC viability and T cell immunogenicity, and indeed that many studies investigating the effect of technical variation during PBMC processing have reported contradictory results (**Supplementary Table 1**). The objective of this review is to briefly discuss the key research that has demonstrated technical variation during this process can profoundly influence cellular viability and immunogenicity (**Figure 1**), highlight the challenges of comparing immunogenicity across samples exposed to variant protocols, and emphasize the need for stringent protocol standardization.

## Sources of variation in cellular viability and immunogenicity

### Sample collection

#### Choice of anticoagulant

When collecting peripheral blood for PBMC isolation, clinically-convenient anticoagulant-lined vacuum-tubes are commonly used for venepuncture. Different anticoagulants, typically Ethylenediaminetetraacetic acid (EDTA), heparin, or citrate, serve specific purposes and have advantages and disadvantages relative to others (**Supplementary Table 2**) (33). According to the HANC-SOP, it is mandatory to document the type of anticoagulant used for each sample (31). Use of EDTA rather than heparin has been linked to diminished immunogenicity following PBMC stimulation (34). Conversely, other studies have found no significant change of functionality between EDTA and sodium-heparin isolated PBMCs (35), nor between sodium-heparin and lithium-heparin collection (36). Anticoagulant has been shown to not be associated with PBMC viability (35), however this study did note viability was statistically associated with anticoagulant when the cryopreservation of PBMCs was delayed. Taken together, these studies report a potential connection between anticoagulant and PBMC viability and functionality and highlight the first potential technical pitfall when studying T cells: the absence of standardised or intentional anticoagulant selection.

#### Processing time and temperature

It is generally accepted the post-venepuncture processing time and temperature are critical parameters affecting cellular viability and T cell immunogenicity (35, 37). However, PBMCs are routinely isolated from peripheral blood well beyond 24 hours after venepuncture, especially in clinical trials (26, 38). The HANC-SOP recommends that processing time should not exceed 8 hours (31). Processing delays of 24 hours or more have been associated with reduced cell viability (35), and ambient temperatures less than 22°C reduced PBMC viability and immunogenicity (39). Nucleic acid recovery from whole blood was profoundly reduced following exposure to suboptimal processing times and temperatures (40). However, these results are challenged by another study which found a 24-hour delay in blood sample processing did not affect the viability of PBMCs, nor the amount of mitogen-induced protein secretion (37). Although conflicting studies exist, discrepancies in the timing and temperature conditions of PBMC processing clearly pose a significant challenge for T cell research. The HANC-SOP requires the collection, processing, and freezing date and time to be documented (31). We propose the ambient temperature should also be reported.

#### PBMC isolation

PBMCs are typically isolated from peripheral blood using density-gradient centrifugation methods, such as Ficoll-Paque, or in clinically-convenient cell preparation tubes (CPTs), including SepMate^™^ and Vacutainer^®^ CPTs (41). The HANC-SOP requires the isolation method and processing technician to be documented (31). Ficoll-processed PBMCs were found to have higher viability when compared to CPT-processed PBMCs (42). Another study reported that differences in viability due to isolation method were detected in one laboratory but not another (43). In contrast, others have reported no significant differences in cell viability and recovery when isolating PBMCs with either Ficoll-Paque or CPTs (35, 44). Immunogenicity has also been associated with isolation method, as PBMCs isolated using Ficoll-Paque were found to secrete lower levels of the cytokine Interferon-gamma (IFN-γ) compared to those isolated with CPTs (41). However, transcriptomic profiles were not found to be influenced by isolation method (44). These results may be confounded by technician experience, which has been estimated to contribute to approximately 60% of the variability of cell recovery (41). The findings of these studies suggest that standardising PBMC isolation procedures and technician training is likely to enhance the reproducibility and reliability of T cell research.

### Cryopreservation

#### Fresh PBMCs

PBMCs are used in immunoassays either immediately (fresh) or following cryopreservation. Cryopreservation can profoundly influence the viability and immunogenicity of T cells. Indeed, the kinetics of cytokine expression, proliferation, cell viability, and immunophenotypes were demonstrated to differ between freshly isolated and matched cryopreserved PBMCs (45). However, many studies have demonstrated minimal post-cryopreservation differences. A multi-site study across nine laboratories was able to recover similar PBMC numbers following cryopreservation without significant loss of viability (46), while others have demonstrated full functionality of cryopreserved T cells (47). Nevertheless, results from these single studies have not significantly influenced typical PBMC cryopreservation protocols, and reporting the status of cells, whether fresh or cryopreserved, is generally expected.

#### Cryopreservation media

The first stage of cryopreservation is typically to suspend PBMCs in a cryoprotective agent, such as dimethylsulfoxide (DMSO) (48). The HANC-SOP specifies PBMCs should be gently resuspended to 10^7^/mL in a 10% DMSO 90% Foetal Calf Serum (FCS) cryopreservation media cooled to 2 to 8°C with continuous swirling (31). The concentration of DMSO has been found to be usually the most important factor determining cellular viability (49) and is generally 10% (50, 51). However, one study has reported PBMC recovery was significantly improved when using 5% DMSO (52). A cell concentration greater than 6x10^6^ PBMC/mL has been associated with improved viability (53) and sera in the cryopreservation media has been found to improve viability (54) and immunogenicity (55). Sera is typically either FCS or ‘normal’ human AB serum, and although human sera may be more physiologically relevant (56), one study reported the use of FCS improved human PBMC viability (55), while another reported no significant difference between the two (57). Other studies have reported only a minimal cell viability (58), or immunogenicity (35, 58) improvement when supplementing cryopreservation media with sera, or noted heightened background immunoreactivity when supplementing with bovine serum albumin (BSA) (57). Gradually resuspending PBMCs in cooled cryopreservation media, such as by a drip-wise method, may improve PBMC viability and immunogenicity by minimising toxic shock or cell membrane damage (48, 59). However, the impact of cryoprotectant addition rate on PBMC viability has not been specifically contrasted, unlike in spermatozoa cryopreservation studies, which have yielded inconsistent findings (60, 61). Cooling the cryopreservation media to 4°C has been associated with preserving T cell immunogenicity (62), although others have shown that cooling temperature did not show any significant effects (49). Taken together, these studies demonstrate that cryopreservation media can significantly influence T cell viability and immunogenicity.

#### Cooling rate

Once cells are suspended in the cryopreservation media, they must be cooled to their storage temperature, generally aiming to reduce cell temperature by -1°C min^-1^. Cryopreservation of highly concentrated PBMCS using an automated controlled-rate freezer is the gold-standard, having been found to enhance T cell activation (63). However, typically, cryopreservation is achieved with two-stage cooling, where cells are cooled in ultra-low freezers (ULFs) to -80°C, then in vapor phase liquid nitrogen (LN_2_) to below -150°C in commercial freezing containers, such as a Mr. Frosty^™^. The HANC-SOP requires samples to be immediately frozen in commercial controlled rate freezing containers, first in ULF then LN_2_ to reduce temperature by -1°C min^-1^ (31). Strategies have been developed which avoid cell viability loss during rapid freezing, such as media ice seeding (nucleation) which allowed PBMCs to be cooled at -90°C min^-1^ (64). Another study found cooling rate did not influence viability, as long as thawing rates were high (113°C min^-1^ and 45°C min^-1^) (65). Others have reported no change in cell viability when cells were initially cooled in LN_2_ (66). Despite these reports from single studies, cooling rate is generally accepted to significantly influence T cell viability and immunogenicity. We propose that the freezing container brand should be recorded.

#### Time at -80°C

Cells may be left briefly in ULFs before long-term storage in LN_2_. The HANC-SOP requires samples to be transferred to LN_2_ within 72 hours of freezing (31). Studies have demonstrated storage at -80°C can influence gene expression (67), and significantly reduce PBMC viability and immunogenicity (68). Others have demonstrated a linear relationship between decreasing viability and time on dry ice (-70°C) over 12 weeks (69). Another study found that viability had been lost with as little as 48 hours of storage at -80°C (55). Conversely, others found PBMCs stored on dry ice for three weeks had no significant difference in viability or immunogenicity compared to those immediately cryo-stored (70), while others have reported storing PBMCs on dry ice for three weeks did not reduce the T cell immunogenicity (35). Although some studies found relatively short-term storage at -80°C had a minimal influence on PBMC viability and immunogenicity, the consensus on its potential effect highlights the need to record and standardise the date and time of transfer to LN_2_.

#### Time in cryo-storage

Cells stored in liquid nitrogen can remain viable and functional for very long periods of time and the HANC-SOP states cells may be stored in LN_2_ indefinitely (31). Several studies which directly investigated cryo-storage viability found no clinically significant variation in cellular viability over 15 months (70), or viability and hematopoietic stem cell populations over 60 months (71). However, immunogenicity may be influenced by small but statistically significant variations in the populations of lymphocytes, which have been found to vary following 3-to-6-months of cryo-storage (72, 73). Despite a limited number of studies which have demonstrated an impact of LN_2_ storage duration on PBMC viability or immunogenicity, we nevertheless propose the length of time in cryo-storage should be reported.

#### Exposure to varying temperatures

During long-term cryo-storage, cells are often transiently exposed to briefly varying temperatures as other co-stored aliquots are added or removed from the facility. One study found reduced cell viability and immunogenicity when cyclically exposing cryo-stored PBMCs briefly to room temperature (74). However, another study found viability was not influenced when aliquots of PBMCs went through repeated rounds of temperature cycling (75), while another study investigating gene expression profiles found no significant change of PBMC gene expression following brief but repetitive temperature cycling (67). The HANC-SOP states PBMCs are not to be transferred back to ULF storage (31). Additional studies are required to precisely assess the impact of brief but potentially repetitive temperature fluctuations on the viability and immunogenicity of cryopreserved PBMCs.

#### Shipping

The gold-standard practise to ship cryo-stored PBMCs is with LN_2_-dry shippers, however, dry ice is also used. The HANC-SOP requires PBMCs stored in LN_2_ to be shipped in cold-shippers which maintain LN_2_ temperatures (31). Both viability and immunogenicity were influenced by cold-shipping strategy (69). However, one study found that the shipping method influenced viability, but did not influence immunogenicity (35), while another found that viability was not influenced but lymphocyte populations (*i.e.*, the ratio of CD4^+^/CD8^+^ T cells) were affected (76). It is unclear why one population of T cell would be more sensitive to shipping than another. Significant temperature changes during shipping certainly will impact PBMC viability and immunogenicity. However, similarly to the potential fluctuations which can occur during long-term cryo-storage, exposure to varying temperatures during shipping may be brief but recurrent, and more research is required on the effects of smaller or repeated fluctuations.

### Thawing

#### Thaw time and washing

The IMPAACT-SOP calls for cells to be thawed rapidly at 37°C, then added to the thawing media in a drip-wise action (32). When returning cryo-preserved cells to physiological conditions PBMC viability and immunogenicity has been improved with rapid thawing (65, 77), and therefore, rapid thawing is widely recognized as the preferred method to thaw cryo-preserved cells. However, this effect has been found to be minimal in a single study when the cells were cooled at the rate required by the HANC-SOP (-1°C min^−1^) (65). Once cells are thawed, they are routinely immediately washed to minimise contact with the cryoprotectant. However, one study has controversially reported no change in cell viability following either immediate washing or leaving in the water bath for five minutes (78). Cells and washing medium may be combined by swiftly diluting the cells into the thawing media or by adding in a drip-wise action. No difference in the absolute count of live PBMCs was reported when cells were added rapidly (78). While further studies may identify protocols with more refined thawing rates and washing steps, standardising the current SOP would likely improve the reproducibility and reliability of T cell research.

#### Thawing media & sera

The media used during the thawing process can include various salt-balanced and buffered solutions such as phosphate buffered saline (PBS) or culture media, typically RPMI 1640 medium (RPMI) supplemented with additives including FCS. Sera can vary significantly between batches (79), and the HANC group reserve lots of FCS for batched experiments (32). Several studies investigating various combinations of washing medias have found PBMC viability was highest following washing with media including sera (55, 78), and noted improved cell viability (55, 78), and functionality (80), when thawing with media at a temperature of 37°C rather than 4°C. No significant difference in viability was observed when thawing cells in either 50mL or 15mL of thawing media (55). Collectively, these studies indicate that both thawing media and sera significantly affect T cell viability and immunogenicity, underscoring the importance of standardizing these components to the greatest extent feasible. The IMPAACT-SOP requires the use of 10% approved lot FCS in RPMI (32), we furthermore recommend the media is warmed to 37°C. The optimal sera for human T cell assays are human sera, which although impractical, would ideally be autologously matched (26). FCS is a popular alternative, especially in clinical trials due to its greater consistency, scalability, and cost efficiency.

#### Nuclease digestion

To prevent cell clumping due to nucleic material released from lysed cells, post-thawed and washed cells may be incubated with a nuclease. The use of a DNase endonuclease was reported to have little effect on cell morphology, function, or viability (81). However, a flow cytometry-based study found changes in cell populations with varying forward and side scatter profiles following DNase treatment (82), but no detectable changes in cell viability, expression of standard lymphocyte surface markers, nor intracellular cytokine expression. Benzonase is another commonly used nuclease which allows efficient degradation of all types of DNA and RNA. The use of Benzonase^®^ during PBMC processing has been reported to not influence T cell immunogenicity (83). The IMPAACT-SOP includes the optional inclusion of Benzonase^®^ during the first wash (32), as the enzyme’s properties suggest that it could potentially influence PCR outcomes (84). Further research will be required to provide clearer guidelines for the use of nuclease digestion; however, as no study has demonstrated Benzonase^®^ influences PCR outcomes when used during PBMC thawing, we therefore recommend the routine inclusion of Benzonase^®^.

#### Pre-culture (rest)

Post-thawing, cells can be experimented upon immediately or undergo preculture (or resting). The IMPAACT-SOP includes an optional rest in culture for 14-18 hours (32). Overnight resting has been found to be optimal to increase the immunogenicity of PBMCs (85), while even 1 hour of pre-culture can replicate the surface marker expression of fresh cells (86). However, others have reported preculture had no statistically significant influence on PBMC immunogenicity (87). The concentration of cells during the rest period has also been found to influence cellular immunoreactivity. Short term pre-culture of PBMCs at high concentrations has been found to improve immunogenicity (19), and others reported the immunogenic response varied relative to PBMC preculture concentration (10). Longer term incubation, upwards of 48 hours in extremely high densities (1×10^7^ cells/mL), has been reported to greatly improve the immunogenicity of CD8^+^ T cell responses (14), without influencing viability. The results from these combined studies demonstrate that pre-culture conditions, especially cellular density, can significantly influence T cell immunogenicity, and therefore, we propose that the rest period and cell density should be standardised and recorded.

### Cell culture

#### Cell concentration

During functional immunoassays cell density is typically between 1-4×10^6^ cells/mL to facilitate inter-cellular contact and antigen presentation, which is a concentration not expected to influence cell viability (10, 88). The immunogenic response of PBMCs is profoundly influenced by the concentration of cells in the stimulation reaction, particularly when stimulating with weakly immunoreactive antigenic peptides (10). More highly reactive stimulants are also influenced by cell concentration, with one study determining a PBMC concentration of 2.5×10^6^ cells/mL was optimal to detect cytokine responses following mitogen stimulation (89). There are, however, a relatively limited number of studies which have optimised stimulation cell concentrations. Such optimisation may be impractical, particularly when considering experiments involving antigenic T cell peptide epitopes which may involve thousands of stimulatory conditions. Nevertheless, standardising and reporting cell density during culture is generally expected.

#### Stimulant concentration and solvent

Stimulant concentration is a critical determinant of T-cell functionality. For example, increasing the concentration of antigen is recognised to generally increase the number of IFN-γ^+^ PBMCs (14). Titrated antigen experiments have identified ranges between 1 μg/ml (90) to 10 μg/ml (36) as optimal for MHC-class I peptide epitope-induced immunogenicity. However, a donor-specific effect on the optimal antigen stimulant concentration to induce immunoreactivity has been described (36). While these studies cumulatively confirm that, as expected, stimulant concentration does influence T cell immunoreactivity, there are limited studies optimising stimulant concentrations. Identifying universally optimal stimulant concentrations is impractical, especially for T cell peptide epitope screening studies. Lyophilized peptide epitopes are typically resuspended in DMSO, which has several key advantages and disadvantages relative to other common solvents (**Supplementary Table 3**) (91). Even relatively low concentrations of DMSO in cell culture have been found to induce changes to cellular phenotypes (92), with as little as 0.25% DMSO influencing immunogenicity (93), and marginal toxicity reported at 2-5% DMSO (93). While reporting the stimulant concentration is common practise, we further recommend reporting the concentration and type of solvent in the stimulation.

#### Stimulation time

The length of time cells are incubated in the presence of the stimulant can influence the number and intensity of responsive cells. Six-hour long incubations are frequently reported (94, 95), however, longer incubations are also common (96). Overnight incubations have been reported to increase antigen immunogenicity (97). The optimal timepoint to measure mitogen stimulations has been reported to be between 72 and 96 hours (98). Similarly, kinetic studies investigating whole-blood stimulations found 72 hours as optimal for mitogen stimulants (99). While antigenic peptide stimulation time length has been found to not decrease cell viability, longer mitogen stimulations have been associated with increased numbers of non-viable cells (45). Interestingly, a recent study over a 12 hour time course noted peak cytokine mRNA expression occurred between 3-6 hours post peptide-epitope stimulation, and occurred in a peptide- and donor-specific fashion (95).

## Discussion

Taken together these studies demonstrate that technical variation during PBMC collection, cryopreservation, thawing, and culture may profoundly impact cell viability and immunogenicity. Notably, many of the conclusions of these studies are contradictory, suggesting the influence and interaction of underreported factors. Although several of the results discussed in this review are derived from single studies, these results from well-controlled, high-quality studies nevertheless underscore the importance of maintaining stringently consistent protocols and reporting guidelines when analysing human T cells. Therefore, we propose the use of the HANC’s-SOP when collecting and cryopreserving PBMCs (31); and the IMPAACT networks-SOP when thawing PBMCs (32). Furthermore, we have made additional recommendations to standardise the protocol during PBMC collection, storage, thawing, and culture (**Table 1**).

**Table 1:** Standardised protocol for the collection, cryopreservation, thawing and culturing of human PBMCs for T cell studies.

Major sources of technical variation	HANC and IMPAACT protocol instructions	Further recommendations
Collection		
Anticoagulation	The type of anticoagulant must be recorded*	
Processing Time & Temperature	The collection, processing, and freezing date and time must be recorded*	The ambient temperature should be recorded
Isolation Method	The processing method and processing technician must be recorded*	
Cryopreservation		
Cryopreservation Media	10% DMSO in FCS cryopreservation media cooled to 2 to 8°C must be used*	
Cooling Rate	Immediately freeze in a ULF using a commercial controlled-rate freezing container*	Commercial product should be recorded
Time at -80°C	Transfer to LN_2_ within 72 hours of freezing*	The date and time of transfer to LN_2_ should be recorded
Time in Cryo-storage	Frozen PBMC samples can be stored safely in vapor phase LN_2_ indefinitely*	The date and time of transfer from LN_2_ should be recorded
Exposure to Varying Temperatures	Do not transfer back to ULF*	
Shipping	All transfers must be maintained in LN_2_ *	
Thawing		
Thaw Time and Washing	Thaw cells rapidly at 37°C, added in a drip-wise action †	
Thawing Media & Sera	Wash in RPMI 10% FCS thawing media †	Warm media to 37°C
Nuclease digestion	Optional inclusion of Benzonase^®^ during the first wash †	Benzonase^®^ use should be routine and reported
Preculture	Cells may be optionally rested in culture for 14-18 hours †	The rest period and cell density should be recorded
Culturing		
Cell concentration	Reporting cell density is generally expected.	
Stimulant concentration	Reporting stimulant concentration is generally expected	The solvent type and concentration should be reported
Stimulation Time	Reporting the stimulation time is generally expected	

*The Office of HIV/AIDS Network Coordination (HANC)-SOP; †International Maternal Pediatric Adolescent AIDS Clinical Trials (IMPAACT)-SOP, LN2: vapour phase liquid nitrogen (-180°C), ULF, Ultra-low freezer (-80°C); SOP, standard operating procedure; DMSO, Dimethyl sulfoxide; FCS, Foetal Calf Serum.

The biological mechanisms underlying variations in cell viability and immunogenicity during PBMC processing are complex and incompletely understood. The various reagents used during processing may significantly influence T cells by impacting cellular integrity, agonistically or antagonistically influencing cellular activation, or changing media chemistry. For example, the anticoagulant EDTA can impair T cell activation by disrupting cellular calcium levels (100), while heparin may interfere with cell-to-cell interactions (101). The more profound influences which have been found following delays in processing and fluctuations in temperature during storage can be attributed to induced metabolic stress which may have activated apoptosis pathways (102, 103). Techniques like density-gradient centrifugation and cryopreservation introduce stress through physical forces (104), and if cryoprotectants are not completely removed after thawing, they may further alter cell functions (105). This complexity highlights the critical need for strict standardisation and detailed documentation in T cell research to avoid the technical pitfalls when collecting, cryopreserving, thawing, and stimulating human T-cells.

The development of PBMC processing SOPs by HANC and its affiliated members such as IMPAACT were driven by an awareness that technical variation during this critical experiment window may reduce the reproducibility of experimental findings. There is a growing concern that the social, behavioural, and biomedical sciences are facing a ‘reproducibility crisis’ (106), as many influential published findings have failed reproducibility testing (107, 108). Indeed, a relatively recent large meta-analysis proposed that, at best, around 50% of preclinical biomedical research was reproducible (109). The cause of this low reproducibility is likely complex, ranging from poor statistical literacy (110) and noise discovery (111), to unconscious or conscious bias induced by a pressure to publish (112). While a low rate of reproducibility is certainly not ideal, it has been argued that some irreproducibility is expected (113), even potentially beneficial (114), when cutting-edge science is investigating competing hypotheses. Nevertheless, there is a growing appreciation that insufficient communication of experimental methods is a major contributing factor (115–118). We expect that strict adherence to HANC’s and HANC affiliates SOPs during PBMC collection, cryopreservation, thawing, and culture will greatly improve the replicability of human T cell research.

## Author contributions

DB: Writing – original draft, Writing – review & editing. CM: Writing – review & editing. DD: Writing – review & editing.
