# Supplementary material for: Technical pitfalls when collecting, cryopreserving, thawing, and stimulating human T-cells
Source: Front Immunol. 2024 May 15;15:1382192. doi: 10.3389/fimmu.2024.1382192 (PMC11133553; doi:10.3389/fimmu.2024.1382192)
Supplement: Supplementary file 1 [file DataSheet_1.docx]

**Supplementary Tables:**

| **Supplementary Table 1. Representative studies investigating PBMC collection, storage, thawing and culture.** | | | | |
| --- | --- | --- | --- | --- |
|  | Viability | | Immunogenicity | |
|  | Influenced | Not Influenced or minor | Influenced | Not Influenced or minor |
| Collection | | | | |
| Anticoagulation | Not directly associated with viability (1) | Associated with viability when processing was delayed (1) | Use of EDTA linked to reduced immunogenicity (2) | No significant changed between EDTA or sodium-heparin (1);  No significant changed between sodium-heparin and lithium- heparin (3) |
| Processing Time | Processing delay greater than 24 hours reduced viability (1);  Temperature lower than 22oC reduced viability (4) | A 24-hour delay did not influence viability (5) | Temperature lower than 22oC reduced immunogenicity (4)  RNA was significantly degraded after 24 hours (6) | A 24-hour delay did not influence immunogenicity (5) |
| Isolation Method | Ficoll-processed PBMCs had higher viability when compared to CPT-processed PBMCs (7);  Differences were found to be laboratory specific (8) | No significant difference Ficoll-processed PBMCs when compared to CPT-processed PBMCs (1, 9) | Ficol plaque PBMCs secreted lower levels of IFN-γ when compared to CPT-processed PBMCs (10) | Transcriptomic profiles were not influenced by isolation method (9). |
| Storage | | | | |
| Fresh PBMCs | Cell viability differed between freshly isolated and matched cryopreserved PBMCs (11). | A multi-site study across nine laboratories was able to recover similar PBMC numbers following cryopreservation without significant loss of viability (12) | The kinetics of cytokine expression, proliferation differed between freshly isolated and matched cryopreserved PBMCs (11). | Full functionality was demonstrated of cryopreserved T cells (13) |
| Cryopreservation Media | The concentration of DMSO was found to be the most important factor determining cellular viability (14).  PBMC recovery was significantly improved when using 5% DMSO (15)  A cell concentration greater than 6x10^6^ PBMC/mL has been associated with improved viability (16)  Sera in the cryopreservation media has been found to improve viability (17)  FCS improved human PBMC viability (18) | No significant difference between FCS and human sera (19)  Only minimal improvement in cellular viability 2011 (20) | Sera in the cryopreservation media has been found to improve immunogenicity (18)  Background immunoreactivity when supplementing with BSA (19)  Cooling the cryopreservation media to 4°C has been associated with preserving T cell immunogenicity (21) | Only minimal improvement in immunogenicity(1) (20)  Cooling the media had not significant effect (14). |
| Cooling rate | The cooling rate did not influence viability, as long as thawing rates were high (113°C min^-1^ and 45°C min^-1^) (22)  PBMCs cooled at -90°C min^-1^ did not have viability loss (23) | No change in cell viability when cells were initially cooled in LN_2_ (24) | Cryopreservation of highly concentrated PBMCS using an automated controlled-rate freezer enhanced T cell activation (25) |  |
| Time at -80°C | Storage at -80°C can influence gene expression (26)  viability had been lost with as little as 48 hours of storage at -80°C (18)  a linear relationship between decreasing viability and time on dry ice (-70°C) over 12 weeks (27).    Storage at -80°C significantly reduced PBMC viability (28) | PBMCs stored on dry ice for three weeks had no significant difference in viability compared to those immediately cryo-stored (29) | Storage at -80°C significantly reduced PBMC immunogenicity (28) | PBMCs stored on dry ice for three weeks did not reduce T cell immunogenicity (1).  PBMCs stored on dry ice for three weeks had no significant difference in immunogenicity compared to those immediately cryo-stored (29) |
| Time in Cryo- Storage |  | No clinically significant variation in cellular viability over 15 months (29)  No clinically significant variation in cellular viability over 60 months (30) | Small but statistically significant variations in the populations of lymphocytes. (31, 32) | No clinically significant variation in cellular immunogenicity over 60 months (30) |
| Varying temperatures | Reduced cell viability when cyclically exposing cryo-stored PBMCs briefly to room temperature (33). | Viability was not influenced when aliquots of PBMCs went through repeated rounds of temperature cycling (34) | Reduced cell immunogenicity when cyclically exposing cryo-stored PBMCs briefly to room temperature (33). | No significant change of PBMC gene expression following brief but repetitive temperature cycling (26). |
| Shipping | Shipping method influenced viability (1)  Viability was influenced by cold-shipping strategy (27) | lymphocyte viability was not affected (35) | Lymphocyte populations were affected (35)  Immunogenicity was influenced by cold-shipping strategy (27) | Shipping method did not influence immunogenicity (1) |
| Thawing | | | | |
| Thaw time | PBMC viability has been improved with rapid thawing (22, 36) | No change in cell viability following either immediate washing or leaving in the water bath for five minutes (37) | PBMC immunogenicity has been improved with rapid thawing (36) |  |
| Thawing Media & Sera | Viability was highest following washing with media including sera (18, 37), |  | Immunogenicity was highest when washing with media including sera (38) |  |
| Nuclease digestion |  | DNase endonuclease was reported to have little effect on cell viability (39).  found changes in cell populations with varying forward and side scatter profiles following DNase treatment (40) |  | DNase endonuclease was reported to have little effect on cell function (39).  Benzonase during PBMC processing has been reported to not influence T cell immunogenicity (41) |
| Preculture |  |  | Overnight resting has been found to be optimal to increase the immunogenicity of PBMCs (42)  1 hour of pre-culture can replicate the surface marker expression of fresh cells (43).  Short term pre-culture of PBMCs at high concentrations has been found to improve immunogenicity (44)  Longer term incubation, upwards of 48 hours in extremely high densities (1×10^7^ cells/mL), has been reported to greatly improve the immunogenicity of CD8^+^ T cell responses (45) | Preculture had no statistically significant influence on PBMC immunogenicity (46) |
| Culturing | | | | |
| Cell concentration |  | 1-4×10^6^ cells/mL is a concentration not expected to influence cell viability (47, 48) | The immunogenic response of PBMCs is profoundly influenced by the concentration of cells in the stimulation reaction, particularly when stimulating with weakly immunoreactive antigenic peptides (48)  PBMC concentration of 2.5×10^6^ cells/mL was optimal to detect cytokine responses following mitogen stimulation (49). |  |
| Antigen concentration |  | Marginal PBMC toxicity reported at 2-5% DMSO (50). | Increasing the concentration of antigen is recognised to generally increase the number of IFN-γ^+^ PBMCs (45)  Titrated antigen experiments have identified ranges between 1 μg/ml (51) to 10 μg/ml (3) as optimal for MHC-class I peptide epitope-induced immunogenicity  A donor-specific effect on the optimal antigen stimulant concentration to induce immunoreactivity has been described (3).  Relatively low concentrations of DMSO in cell culture have been found to induce changes to cellular phenotypes (52).  As little as 0.25% DMSO influences immunogenicity (50) |  |
| Stimulation Time |  | Antigenic peptide stimulation time length has been found to not decrease cell viability, (11). | Overnight incubations have been reported to increase antigen immunogenicity (53).  The optimal timepoint to measure mitogen stimulations has been reported to be between 72 and 96 hours (54)  kinetic studies investigating whole-blood stimulations found 72 hours as optimal for mitogen stimulants (55).  peak cytokine mRNA expression occurred between 3-6 hours post peptide-epitope stimulation, and occurred in a peptide- and donor-specific fashion (56). |  |
|  | | | | |

| **Supplementary Table 2. Advantages and disadvantages of anticoagulants used during venepuncture to isolate PBMCs for immune phenotyping and functional immunoassays.** | | | | |
| --- | --- | --- | --- | --- |
| Anticoagulant | Blood Tube Lid | Typical Use | Advantage | Disadvantage |
| K2 Potassium Salt of EDTA | Purple | Haematological examinations | - Preserves cell morphology | - Calcium chelation may inhibit cytokine production. - Overuse may lead to cell shrinkage |
| Sodium or Lithium Heparin | Green | White blood cell analysis | - Minimal cell morphology alteration | - Tends to cause white blood cell clumping. - May inhibit PCR |
| Routinely used anticoagulants | | | | |
| Less commonly used anticoagulants | | | | |
| Sodium Citrate | Blue | Coagulation and platelet function testing | - Reversable coagulation - Preserves the coagulation factors for study | - Changes blood chemistry (pH, ionic concentration) - Dilutes sample |
| Acid Citrate Dextrose | Yellow | Whole blood and erythrocyte survival, blood storage | - Maintains cell viability | - Changes blood chemistry (pH, ionic concentration) - Dilutes sample |
| EDTA: Ethylenediaminetetraacetic acid | | | | |

| **Supplementary Table 3. The advantages and disadvantages of representative solvents available for resuspension of lyophilized peptides.** | | |
| --- | --- | --- |
| Solvent | Advantage | Disadvantage |
| DMSO | - Solubility of both polar and non-polar peptides. - Relatively low reactivity. - Protects peptides from degradation that would otherwise occur in H_2_O - Miscible with cell culture media. | - Toxicity - Not biologically inert. - Can penetrate skin and cell walls relatively rapidly. - Not universally effective solubility |
| Ethanol | - Moderate polarity allows resuspension of a range of peptides. - Lower toxicity than DMSO. - Protects peptides from degradation that would otherwise occur in H_2_O. - Miscible with cell culture media | - Toxicity - May denature sensitive peptides. - Solubility of non-polar peptides is low. |
| H_2_O | - Biocompatibility as water is non-toxic. - More closely mimics physiological conditions. - Effective solvent for polar hydrophilic peptides | - Is unsuitable as a solvent for non-polar hydrophobic peptides. - Microbial contamination as microbes can survive in this solvent. - Stability of some peptides may be low |
| DMSO: Dimethyl sulfoxide | | |

**References**

1. Bull M, Lee D, Stucky J, Chiu YL, Rubin A, Horton H, et al. Defining blood processing parameters for optimal detection of cryopreserved antigen-specific responses for HIV vaccine trials. J Immunol Methods. 2007;322(1-2):57-69.

2. Kumar P, Satchidanandam V. Ethyleneglycol-bis-(beta-aminoethylether)tetraacetate as a blood anticoagulant: preservation of antigen-presenting cell function and antigen-specific proliferative response of peripheral blood mononuclear cells from stored blood. Clin Diagn Lab Immunol. 2000;7(4):578-83.

3. Hoffmeister B, Bunde T, Rudawsky IM, Volk HD, Kern F. Detection of antigen‐specific T cells by cytokine flow cytometry: the use of whole blood may underestimate frequencies. European journal of immunology. 2003;33(12):3484-92.

4. Olson WC, Smolkin ME, Farris EM, Fink RJ, Czarkowski AR, Fink JH, et al. Shipping blood to a central laboratory in multicenter clinical trials: effect of ambient temperature on specimen temperature, and effects of temperature on mononuclear cell yield, viability and immunologic function. Journal of Translational Medicine. 2011;9(1):26.

5. Navas A, Giraldo-Parra L, Prieto MD, Cabrera J, Gómez MA. Phenotypic and functional stability of leukocytes from human peripheral blood samples: considerations for the design of immunological studies. BMC Immunol. 2019;20(1):5.

6. Sarathkumara YD, Browne DJ, Kelly AM, Pattinson DJ, Rush CM, Warner J, et al. The Effect of Tropical Temperatures on the Quality of RNA Extracted from Stabilized Whole-Blood Samples. Int J Mol Sci. 2022;23(18).

7. Ruitenberg JJ, Mulder CB, Maino VC, Landay AL, Ghanekar SA. VACUTAINER® CPT™ and Ficoll density gradient separation perform equivalently in maintaining the quality and function of PBMC from HIV seropositive blood samples. BMC Immunology. 2006;7(1):11.

8. Nilsson C, Aboud S, Karlén K, Hejdeman B, Urassa W, Biberfeld G. Optimal Blood Mononuclear Cell Isolation Procedures for Gamma Interferon Enzyme-Linked Immunospot Testing of Healthy Swedish and Tanzanian Subjects. Clinical and Vaccine Immunology. 2008;15(4):585-9.

9. Chen H, Schürch CM, Noble K, Kim K, Krutzik PO, O’Donnell E, et al. Functional comparison of PBMCs isolated by Cell Preparation Tubes (CPT) vs. Lymphoprep Tubes. BMC Immunology. 2020;21(1):15.

10. Hendrika W. Grievink TL, Cornelis Kluft, Matthijs Moerland, and Karen E. Malone. Comparison of Three Isolation Techniques for Human Peripheral Blood Mononuclear Cells: Cell Recovery and Viability, Population Composition, and Cell Functionality. Biopreservation and Biobanking. 2016;14(5):410-5.

11. Jeurink PV, Vissers YM, Rappard B, Savelkoul HFJ. T cell responses in fresh and cryopreserved peripheral blood mononuclear cells: Kinetics of cell viability, cellular subsets, proliferation, and cytokine production. Cryobiology. 2008;57(2):91-103.

12. Reimann KA, Chernoff M, Wilkening CL, Nickerson CE, Landay AL. Preservation of Lymphocyte Immunophenotype and Proliferative Responses in Cryopreserved Peripheral Blood Mononuclear Cells from Human Immunodeficiency Virus Type 1-Infected Donors: Implications for Multicenter Clinical Trials. Clinical Diagnostic Laboratory Immunology. 2000;7(3):352-9.

13. Kreher CR, Dittrich MT, Guerkov R, Boehm BO, Tary-Lehmann M. CD4+ and CD8+ cells in cryopreserved human PBMC maintain full functionality in cytokine ELISPOT assays. Journal of Immunological Methods. 2003;278(1):79-93.

14. Nazarpour R, Zabihi E, Alijanpour E, Abedian Z, Mehdizadeh H, Rahimi F. Optimization of Human Peripheral Blood Mononuclear Cells (PBMCs) Cryopreservation. Int J Mol Cell Med. 2012;1(2):88-93.

15. Kaiser D, Otto NM, McCallion O, Hoffmann H, Zarrinrad G, Stein M, et al. Freezing Medium Containing 5% DMSO Enhances the Cell Viability and Recovery Rate After Cryopreservation of Regulatory T Cell Products ex vivo and in vivo. Frontiers in Cell and Developmental Biology. 2021;9.

16. Hope CM, Huynh D, Wong YY, Oakey H, Perkins GB, Nguyen T, et al. Optimization of Blood Handling and Peripheral Blood Mononuclear Cell Cryopreservation of Low Cell Number Samples. Int J Mol Sci. 2021;22(17).

17. Bogoslovsky T, Wang D, Maric D, Scattergood-Keepper L, Spatz M, Auh S, et al. Cryopreservation and Enumeration of Human Endothelial Progenitor and Endothelial Cells for Clinical Trials. J Blood Disord Transfus. 2013;4(5).

18. Disis ML, dela Rosa C, Goodell V, Kuan L-Y, Chang JCC, Kuus-Reichel K, et al. Maximizing the retention of antigen specific lymphocyte function after cryopreservation. Journal of Immunological Methods. 2006;308(1):13-8.

19. Filbert H, Attig S, Bidmon N, Renard BY, Janetzki S, Sahin U, et al. Serum-free freezing media support high cell quality and excellent ELISPOT assay performance across a wide variety of different assay protocols. Cancer Immunology, Immunotherapy. 2013;62(4):615-27.

20. Anja Germann JCS, Beatrice Kemp-Kamke, Heiko Zimmermann, and Hagen von Briesen. Standardized Serum-Free Cryomedia Maintain Peripheral Blood Mononuclear Cell Viability, Recovery, and Antigen-Specific T-Cell Response Compared to Fetal Calf Serum-Based Medium. Biopreservation and Biobanking. 2011;9(3):229-36.

21. TREE TIM, ROEP BO, PEAKMAN M. Enhancing the Sensitivity of Assays to Detect T Cell Reactivity: The Effect of Cell Separation and Cryopreservation Media. Annals of the New York Academy of Sciences. 2004;1037(1):26-32.

22. Baboo J, Kilbride P, Delahaye M, Milne S, Fonseca F, Blanco M, et al. The Impact of Varying Cooling and Thawing Rates on the Quality of Cryopreserved Human Peripheral Blood T Cells. Scientific Reports. 2019;9(1):3417.

23. Huang Z, Liu W, Liu B, He X, Guo H, Xue S, et al. Cryopreservation of human T lymphocytes under fast cooling with controlled ice nucleation in cryoprotective solutions of low toxicity. Cryobiology. 2021;103:92-100.

24. Mrowiec ZR, Fernandez-DeLeon M, Marchioni M, Cieszynska B. Comparison of Controlled vs Non-Controlled Rate Freezing of Umbilical Cord Blood Units. Blood. 2004;104(11):5011-.

25. Buhl T, Legler TJ, Rosenberger A, Schardt A, Schön MP, Haenssle HA. Controlled-rate freezer cryopreservation of highly concentrated peripheral blood mononuclear cells results in higher cell yields and superior autologous T-cell stimulation for dendritic cell-based immunotherapy. Cancer Immunology, Immunotherapy. 2012;61(11):2021-31.

26. Yang J, Diaz N, Adelsberger J, Zhou X, Stevens R, Rupert A, et al. The effects of storage temperature on PBMC gene expression. BMC Immunology. 2016;17(1):6.

27. Weinberg A, Song LY, Wilkening CL, Fenton T, Hural J, Louzao R, et al. Optimization of storage and shipment of cryopreserved peripheral blood mononuclear cells from HIV-infected and uninfected individuals for ELISPOT assays. J Immunol Methods. 2010;363(1):42-50.

28. Valeri CR, Pivacek LE. Effects of the temperature, the duration of frozen storage, and the freezing container on in vitro measurements in human peripheral blood mononuclear cells. Transfusion. 1996;36(4):303-8.

29. Weinberg A, Song LY, Wilkening C, Sevin A, Blais B, Louzao R, et al. Optimization and limitations of use of cryopreserved peripheral blood mononuclear cells for functional and phenotypic T-cell characterization. Clin Vaccine Immunol. 2009;16(8):1176-86.

30. McCullough J, Haley R, Clay M, Hubel A, Lindgren B, Moroff G. Long-term storage of peripheral blood stem cells frozen and stored with a conventional liquid nitrogen technique compared with cells frozen and stored in a mechanical freezer. Transfusion. 2010;50(4):808-19.

31. Li B, Yang C, Jia G, Liu Y, Wang N, Yang F, et al. Comprehensive evaluation of the effects of long-term cryopreservation on peripheral blood mononuclear cells using flow cytometry. BMC Immunology. 2022;23(1):30.

32. Tollerud DJ, Brown LM, Clark JW, Neuland CY, Mann DL, Pankiw-Trost LK, et al. Cryopreservation and long-term liquid nitrogen storage of peripheral blood mononuclear cells for flow cytometry analysis: effects on cell subset proportions and fluorescence intensity. J Clin Lab Anal. 1991;5(4):255-61.

33. Germann A, Oh Y-J, Schmidt T, Schön U, Zimmermann H, von Briesen H. Temperature fluctuations during deep temperature cryopreservation reduce PBMC recovery, viability and T-cell function. Cryobiology. 2013;67(2):193-200.

34. Preliminary Report: Evaluation of Storage Conditions and Cryococktails during Peripheral Blood Mononuclear Cell Cryopreservation. Cell Preservation Technology. 2007;5(4):189-204.

35. Posevitz-Fejfár A, Posevitz V, Gross CC, Bhatia U, Kurth F, Schütte V, et al. Effects of Blood Transportation on Human Peripheral Mononuclear Cell Yield, Phenotype and Function: Implications for Immune Cell Biobanking. PLOS ONE. 2014;9(12):e115920.

36. Xu Y, Zou Q, Gao F, Wang D, Xue S, Lin H, et al. Effect of Warming Process on the Survival of Cryopreserved Human Peripheral Blood Mononuclear Cells. Biopreserv Biobank. 2021;19(4):318-23.

37. Hønge BL, Petersen MS, Olesen R, Møller BK, Erikstrup C. Optimizing recovery of frozen human peripheral blood mononuclear cells for flow cytometry. PLoS One. 2017;12(11):e0187440.

38. Ramachandran H, Laux J, Moldovan I, Caspell R, Lehmann PV, Subbramanian RA. Optimal thawing of cryopreserved peripheral blood mononuclear cells for use in high-throughput human immune monitoring studies. Cells. 2012;1(3):313-24.

39. Darzynkiewicz Z, Li X, Gong J. Chapter 2 Assays of Cell Viability: Discrimination of Cells Dying by Apoptosis. In: Darzynkiewicz Z, Paul Robinson J, Crissman HA, editors. Methods in Cell Biology. 41: Academic Press; 1994. p. 15-38.

40. García-Piñeres AJ, Hildesheim A, Williams M, Trivett M, Strobl S, Pinto LA. DNAse treatment following thawing of Cryopreserved PBMC is a procedure suitable for lymphocyte functional studies. Journal of Immunological Methods. 2006;313(1):209-13.

41. Smith JG, Liu X, Kaufhold RM, Clair J, Caulfield MJ. Development and Validation of a Gamma Interferon ELISPOT Assay for Quantitation of Cellular Immune Responses to Varicella-Zoster Virus. Clinical Diagnostic Laboratory Immunology. 2001;8(5):871-9.

42. Santos R, Buying A, Sabri N, Yu J, Gringeri A, Bender J, et al. Improvement of IFNg ELISPOT Performance Following Overnight Resting of Frozen PBMC Samples Confirmed Through Rigorous Statistical Analysis. Cells. 2015;4(1):1-18.

43. Lemieux J, Jobin C, Simard C, Néron S. A global look into human T cell subsets before and after cryopreservation using multiparametric flow cytometry and two-dimensional visualization analysis. Journal of Immunological Methods. 2016;434:73-82.

44. Römer PS, Berr S, Avota E, Na S-Y, Battaglia M, ten Berge I, et al. Preculture of PBMCs at high cell density increases sensitivity of T-cell responses, revealing cytokine release by CD28 superagonist TGN1412. Blood. 2011;118(26):6772-82.

45. Wegner J, Hackenberg S, Scholz C-J, Chuvpilo S, Tyrsin D, Matskevich AA, et al. High-density preculture of PBMCs restores defective sensitivity of circulating CD8 T cells to virus- and tumor-derived antigens. Blood. 2015;126(2):185-94.

46. Kuerten S, Batoulis H, Recks MS, Karacsony E, Zhang W, Subbramanian RA, et al. Resting of Cryopreserved PBMC Does Not Generally Benefit the Performance of Antigen-Specific T Cell ELISPOT Assays. Cells. 2012;1(3):409-27.

47. Lozano-Ojalvo D, Lopez-Fandino R, Lopez-Exposito I. PBMC-Derived T Cells. In: Verhoeckx K, Cotter P, Lopez-Exposito I, Kleiveland C, Lea T, Mackie A, et al., editors. The Impact of Food Bioactives on Health: in vitro and ex vivo models. Cham (CH)2015. p. 169-80.

48. Browne DJ, Brady JL, Waardenberg AJ, Loiseau C, Doolan DL. An Analytically and Diagnostically Sensitive RNA Extraction and RT-qPCR Protocol for Peripheral Blood Mononuclear Cells. Frontiers in Immunology. 2020;11.

49. Sävendahl L, Underwood LE. Decreased Interleukin-2 Production from Cultured Peripheral Blood Mononuclear Cells in Human Acute Starvation*. The Journal of Clinical Endocrinology & Metabolism. 1997;82(4):1177-80.

50. Elisia I, Nakamura H, Lam V, Hofs E, Cederberg R, Cait J, et al. DMSO Represses Inflammatory Cytokine Production from Human Blood Cells and Reduces Autoimmune Arthritis. PLOS ONE. 2016;11(3):e0152538.

51. Kern F, Bunde T, Faulhaber N, Kiecker F, Khatamzas E, Rudawski IM, et al. Cytomegalovirus (CMV) phosphoprotein 65 makes a large contribution to shaping the T cell repertoire in CMV-exposed individuals. The Journal of infectious diseases. 2002;185(12):1709-16.

52. Tunçer S, Gurbanov R, Sheraj I, Solel E, Esenturk O, Banerjee S. Low dose dimethyl sulfoxide driven gross molecular changes have the potential to interfere with various cellular processes. Sci Rep. 2018;8(1):14828.

53. Bourguignon P, Clément F, Renaud F, Le Bras V, Koutsoukos M, Burny W, et al. Processing of blood samples influences PBMC viability and outcome of cell-mediated immune responses in antiretroviral therapy-naïve HIV-1-infected patients. Journal of Immunological Methods. 2014;414:1-10.

54. Listvanova S, Temmerman S, Stordeur P, Verscheure V, Place S, Zhou L, et al. Optimal kinetics for quantification of antigen-induced cytokines in human peripheral blood mononuclear cells by real-time PCR and by ELISA. Journal of Immunological Methods. 2003;281(1):27-35.

55. Godoy-Ramirez K, Franck K, Mahdavifar S, Andersson L, Gaines H. Optimum culture conditions for specific and nonspecific activation of whole blood and PBMC for intracellular cytokine assessment by flow cytometry. Journal of Immunological Methods. 2004;292(1):1-15.

56. Browne DJ, Kelly AM, Brady JL, Doolan DL. A high-throughput screening RT-qPCR assay for quantifying surrogate markers of immunity from PBMCs. Frontiers in Immunology. 2022;13.
